# Clinical, Radiological, and Histopathological Analysis of Lipoleiomyoma: A Retrospective Study From a Tertiary Care Center

**DOI:** 10.7759/cureus.109168

**Published:** 2026-05-19

**Authors:** Nandhana Devi, Barathi Gunabooshanam, Banukeerthana R

**Affiliations:** 1 Pathology, Sri Ramachandra Institute of Higher Education and Research, Chennai, IND

**Keywords:** estrogen, gynecological pathology, histology, lipoleiomyoma, perimenopause

## Abstract

Introduction

Uterine leiomyoma is a benign smooth muscle tumor commonly occurring in women of reproductive age and showing a myriad of morphologies. Variants account for a minority of these tumors. Lipoleiomyoma is a rare variant of leiomyoma composed of mature adipose tissue intricately interspersed within smooth muscle proliferation. This benign neoplasm has shown a proclivity toward perimenopausal and menopausal obese women. Despite its benign nature, clinical gumption is necessary, as its presence may herald coexistent gynecological pathologies, both benign and malignant.

Methodology

This is a retrospective study of 18 patients with histopathologically diagnosed lipoleiomyoma over a five-year period, from January 2021 to December 2025. Retrospective microscopic evaluation was performed on 10% formalin-fixed hysterectomy or myomectomy specimens, along with the acquisition of clinical, radiological, and gross details from patients' records.

Results

A total of 18 patients included in the study varied in age from 25 to 72 years. Of these, 14 were within the perimenopausal age group, whilst two patients were within the reproductive age group. Clinical presentation varied from abnormal uterine bleeding to mass descending per vagina. A few lipoleiomyomas were incidentally detected during evaluation of adnexal cysts. Notably, 10 out of 18 patients were diabetic, whilst four had impaired glucose tolerance. Radiological assessment favored leiomyoma in most cases, whilst a few were reported to have hyperechoic or lipomatous components on ultrasound. Histopathological evaluation of the resected specimens uncovered other coexisting benign and malignant gynecological conditions, such as adenomyosis, endometrial polyp, and cervical adenocarcinoma, respectively.

Conclusion

Given these associations, it is imperative to carefully evaluate patients clinically and pathologically for coexisting gynecological conditions, both benign and malignant, along with metabolic disorders when lipoleiomyoma is detected. This helps avoid the delayed discovery of potentially morbid and fatal diseases.

## Introduction

Uterine leiomyomas are benign smooth muscle tumors commonly occurring in women of reproductive age and showing a myriad of morphologies. Variants account for 10% of these tumors [1]. Lipoleiomyoma is a rare variant of leiomyoma composed of mature adipose tissue intricately interspersed within smooth muscle proliferation. Its incidence has increased from 0.03%-0.2% to 2%, given advancements in imaging and increased awareness [2,3]. This benign neoplasm has shown a proclivity toward perimenopausal and menopausal obese women. Despite its benign nature, clinical gumption is necessary, as its presence may herald coexisting gynecological pathologies, both benign and malignant.

Aims and objectives

This study aimed to retrospectively evaluate the clinical, radiological, and histopathological features, as well as coexisting metabolic and gynecological pathologies, both benign and malignant, in patients with lipoleiomyoma.

## Materials and methods

Study design

This is a retrospective study of 18 histopathologically proven cases of lipoleiomyoma from January 2021 to December 2025 in the Department of Pathology at Sri Ramachandra Institute of Higher Education and Research, Chennai, India.

Study population

This study was done only on archived samples of formalin-fixed, paraffin-embedded tissue blocks from hysterectomy and myomectomy specimens with a histopathological diagnosis of lipoleiomyoma.

Inclusion and exclusion criteria

The inclusion criteria were all hysterectomy and myomectomy specimens histopathologically diagnosed as lipoleiomyoma.

The exclusion criteria were all other leiomyomas not histopathologically proven or reported as lipoleiomyoma, and lipoleiomyoma cases that lacked H&E slides.

Sampling and sample size

All patients with histopathologically proven lipoleiomyoma (convenient sampling) from the LIS data were chosen for this study. A total of 18 formed the sample size.

Retrospective microscopic evaluation was performed on 10% formalin-fixed, surgically resected specimens that were paraffin-embedded and hematoxylin and eosin-stained in the Department of Pathology. Clinical and radiological data were acquired from patients’ records through the laboratory information system. Histopathological and gross details were collected from pathology case files. The parameters studied included age, anatomical location, tumor size, and coexisting gynecological pathologies.

Data analysis

Clinicopathological details were entered into Microsoft Excel software (Microsoft Corp., Redmond, WA, USA) and analyzed. Descriptive statistics (frequencies and percentages) were used to summarize the results.

Ethical approval

Approval was obtained in April 2026 from the Institutional Research Ethics Committee (IEC) of Sri Ramachandra Institute of Higher Education and Research (IEC reference ID CSP-MED/26/APR/127/118) before the commencement of the study.

## Results

A total of 18 patients included in the study varied in age from 25 to 72 years, with an average of 49.8 years. Of these, 14 were within the perimenopausal age group, whilst two patients were within the reproductive age group, the latter being infrequent. Clinical presentation varied from abnormal uterine bleeding to a mass descending per vagina. Postmenopausal bleeding was observed in nine patients. A few (3/18) lipoleiomyomas were incidentally detected while evaluating adnexal cysts. Notably, 10 out of 18 patients were diabetic, whilst four had impaired glucose tolerance.

**Figure 1:**
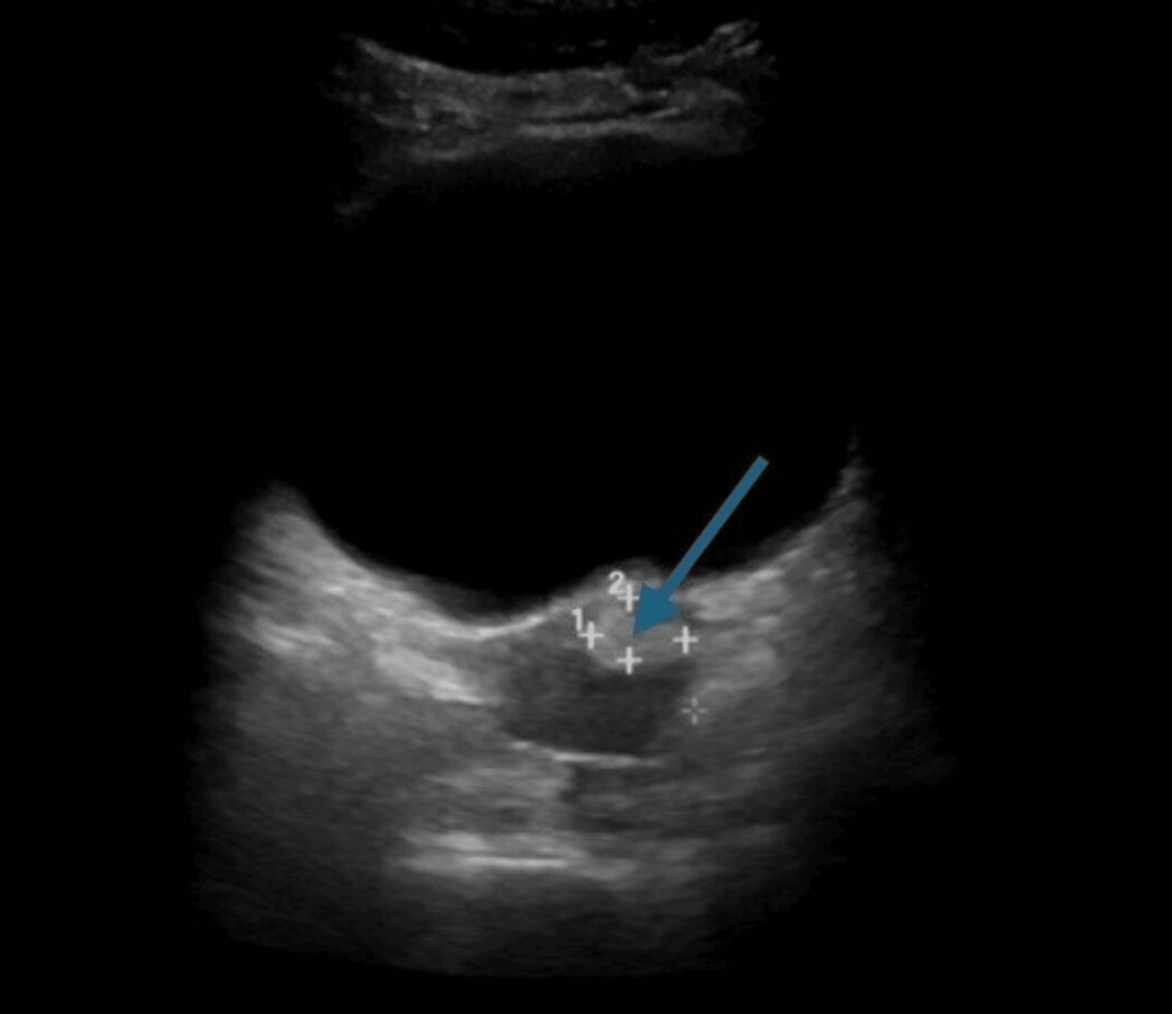
Ultrasound showing a hyperechoic lesion in the anterior myometrial wall (see arrowhead).

Radiological assessment in most cases favored leiomyoma, whilst some (7/18) were reported to have a lipomatous component on ultrasound and MRI. Ultrasound imaging described these lesions as hyperechoic masses (Figure 1).

**Figure 2:**
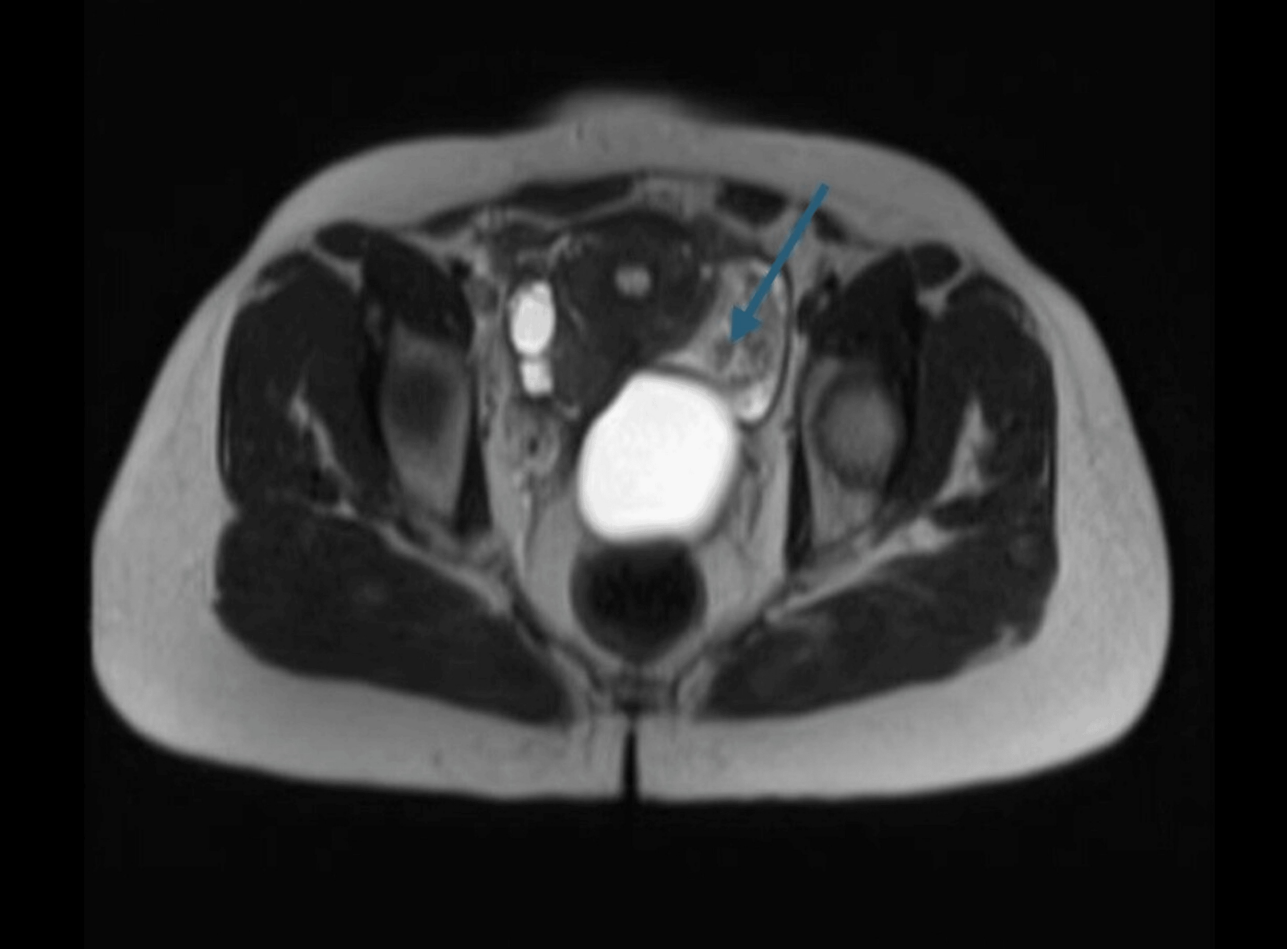
MRI showing lipoleiomyoma as a T1 hyperintense lesion (see arrowhead).

**Figure 3:**
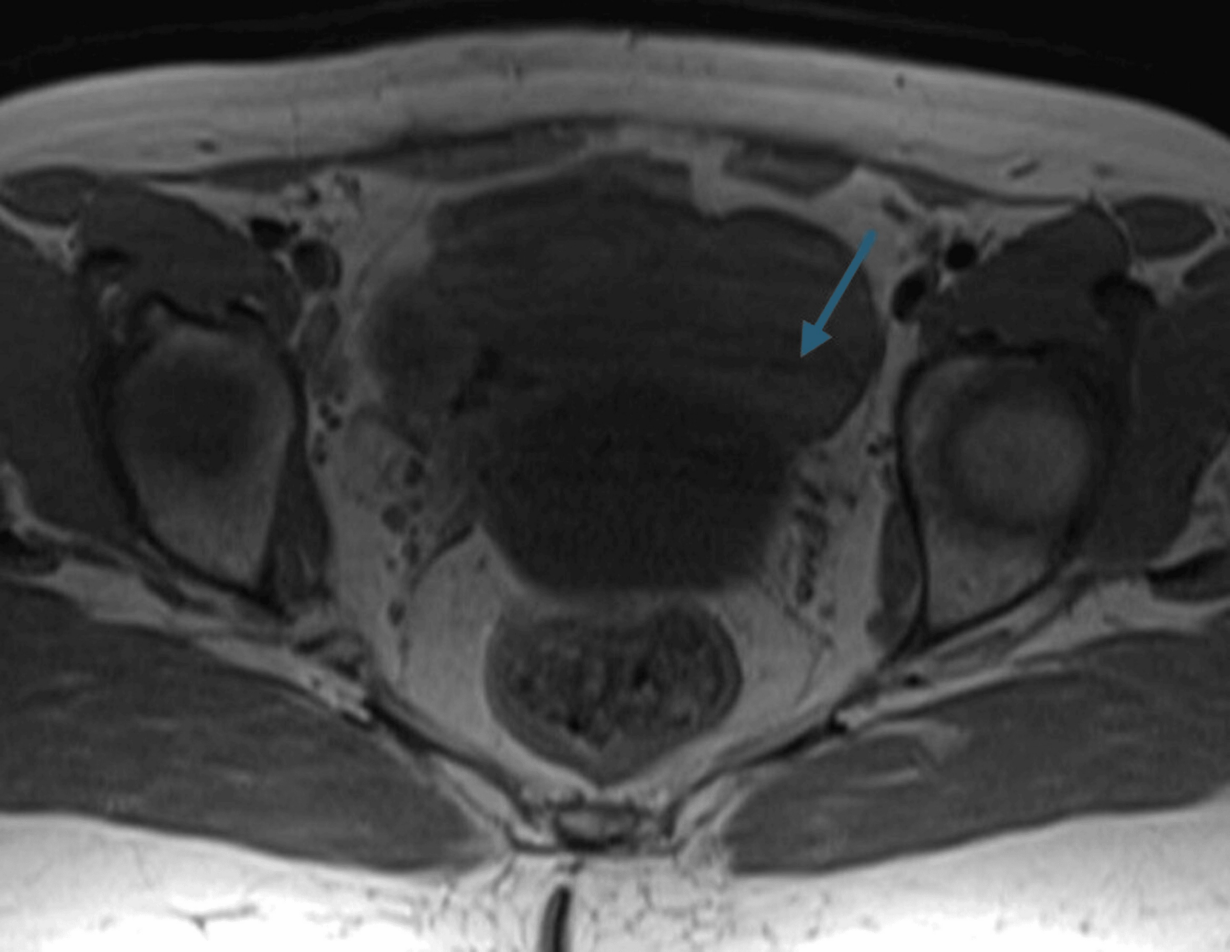
Lipoleiomyoma seen in Figure 2 shows low intensity following fat-suppressed imaging on MRI, confirming the adipocytic component (see arrowhead).

MRI T1 images showed lipoleiomyoma to be hyperintense. Subsequent fat-suppression imaging produced low intensity, confirming the adipocytic component as opposed to hemorrhage or calcification, which can appear similar (Figures 2-3).

**Figure 4:**
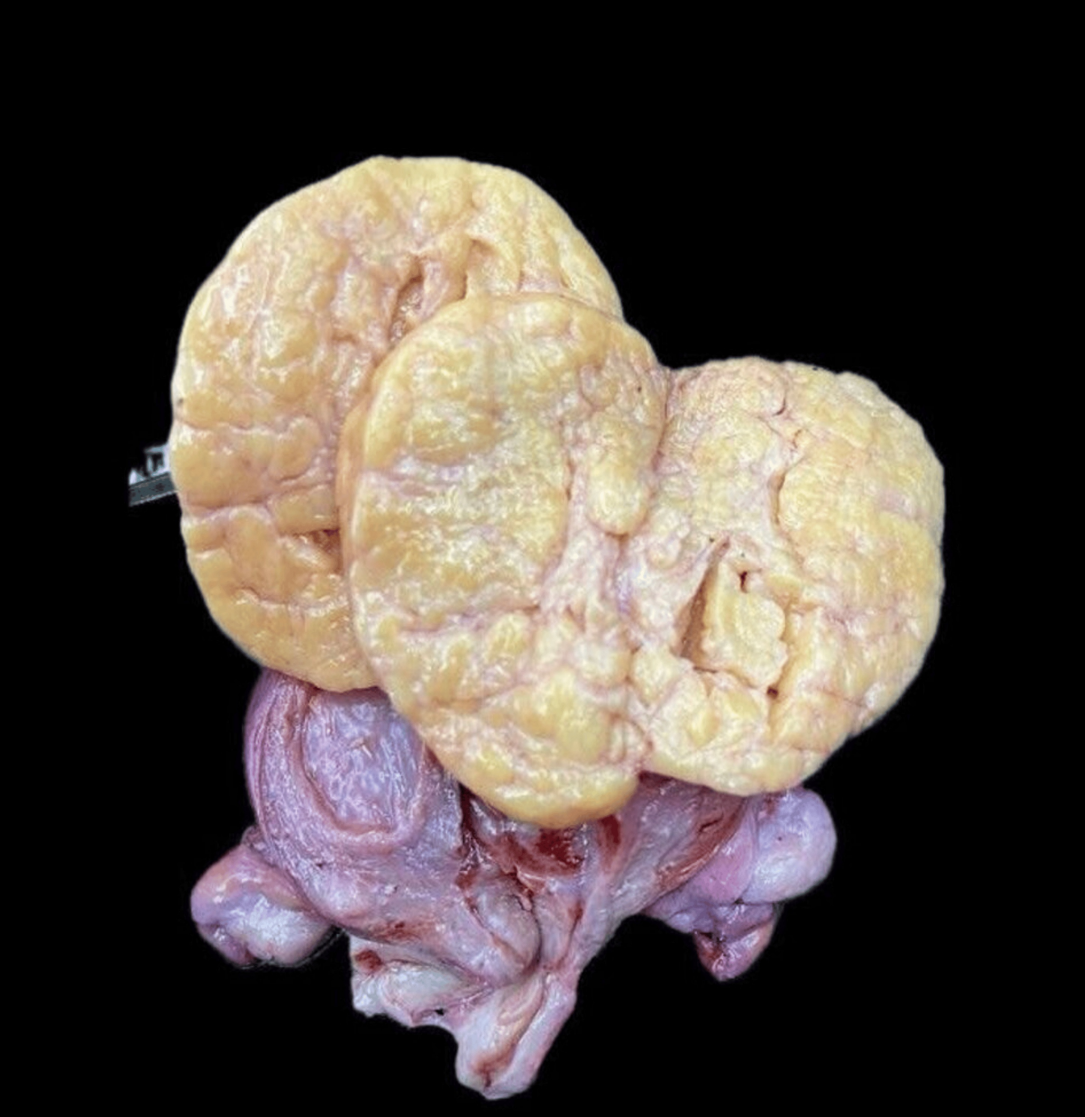
Uterus with cervix showing an intramural, lobulated, yellow mass measuring 9 cm in greatest dimension.

The corpus of the uterus is the most common location in this study group, followed by the cervix. Notably, 15 out of 18 patients underwent hysterectomy, whilst the remaining three underwent fertility-preserving myomectomy. Grossly, the size of these lesions ranged from 1 to 20 cm across the 18 patients, and a lipomatous component was identified in six specimens. These were identified grossly as a yellow to brown, greasy component within a white, firm fibroid showing whorling (Figure 4).

**Figure 5:**
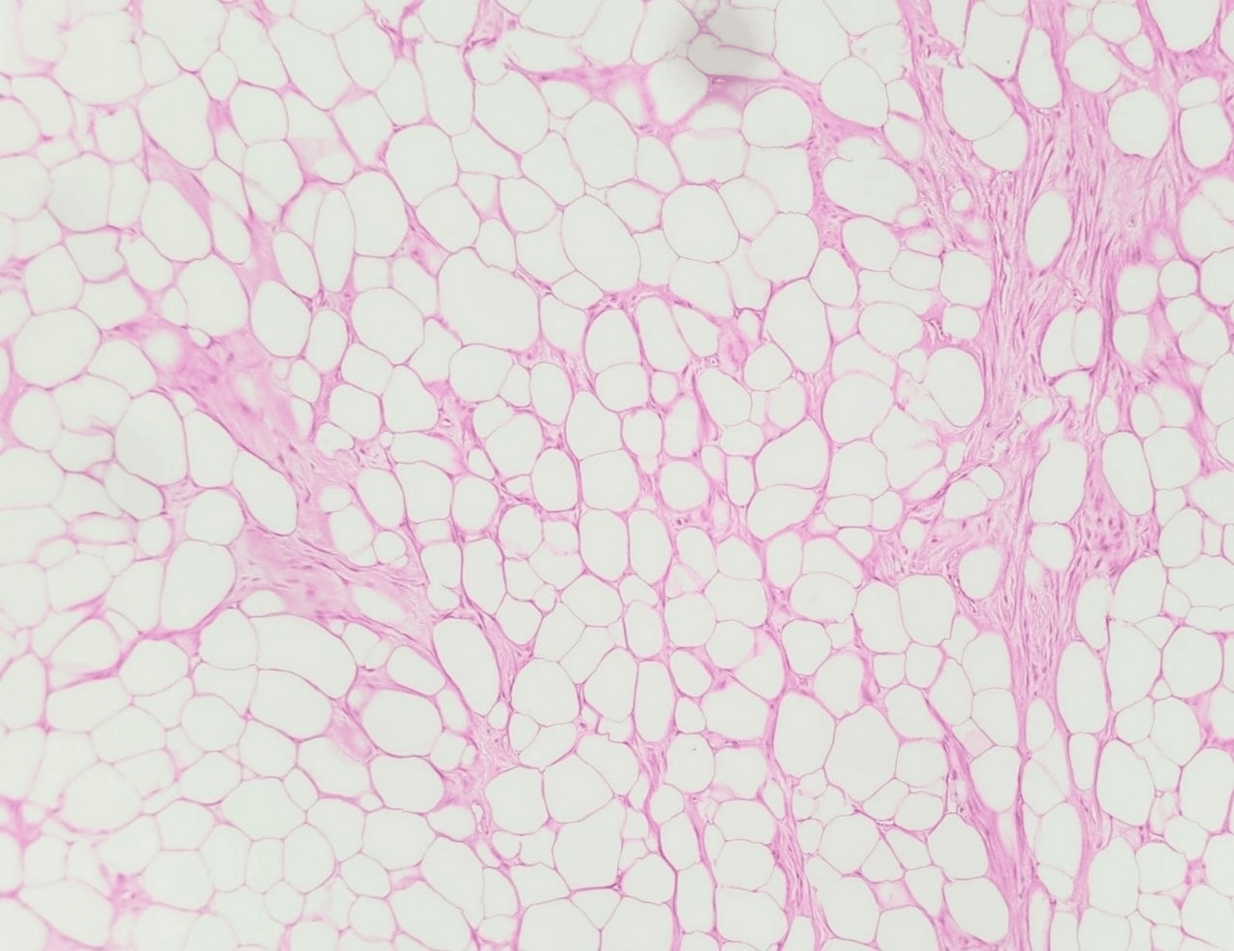
Photomicrograph of lipoleiomyoma showing lobules of mature adipocytes interspersed with fibrous septae and spindle-shaped smooth muscle cells (hematoxylin and eosin stain, 100×).

Microscopically, all lesions were composed of smooth muscle and fibrous tissue arranged in fascicles, intermixed with a variable amount of adipocytes. A few showed hyaline degeneration. None exhibited atypical features such as nuclear atypia, increased mitoses, necrosis, or the presence of lipoblasts (Figure 5).

**Figure 6:**
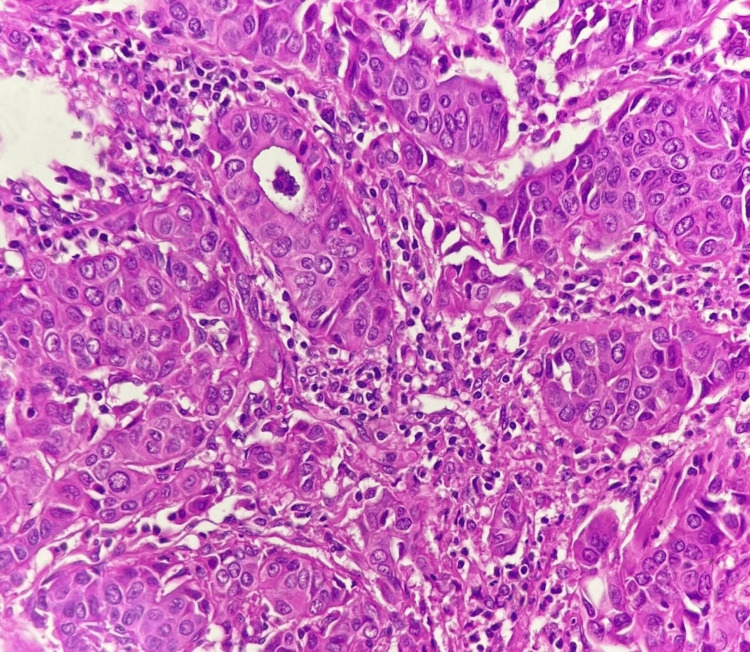
Photomicrograph showing coexisting malignant glands (cervical adenocarcinoma) in the cervix of a 53-year-old postmenopausal woman with uterine lipoleiomyoma (hematoxylin and eosin stain, 400×).

Complete histopathological evaluation of the resected specimens uncovered other coexisting gynecological conditions, both benign and malignant (Figure 6).​​​​

The frequency of the coexisting gynecological conditions has been listed in Table 1.

**Table 1:** Frequency of coexisting gynecological conditions.

Gynecological pathology	Incidence	Percentage
Adenomyosis	6	33%
Mature cystic teratoma	1	5.5%
Serous cyst	2	11.1%
Endometriosis	1	5.5%
Benign endometrial polyp	5	27.7%
Cervical intraepithelial neoplasia	1	5.5%
Adenocarcinoma	1	5.5%

## Discussion

Clinical presentation

Lipoleiomyoma is unique from its parent entity, leiomyoma, in that it occurs in perimenopausal or obese menopausal women, with the median age ranging from 50 to 70 years. It is the most common variant in the aforementioned demograph, peaking in growth after menopause [4-6]. It was first described in 1991 by Meis and Enzinger. Lipomatous tumors of the uterus can be categorized as pure lipomas, lipomas with mesenchymal elements, which include lipoleiomyoma, and the malignant counterpart, liposarcoma [1]. They are commonly found in the corpus of the uterus as intramural or subserosal masses. They can also occur in the cervix, broad ligament, ovary, and retroperitoneum [7,8]. Herein, we have two patients with cervical origin of this pathology.

Patients are generally asymptomatic and are detected incidentally on radiological assessment or intraoperatively. When symptomatic, compressive symptoms such as increased urinary frequency or incontinence, pelvic pain as a corollary of the mass, and abnormal uterine bleeding in perimenopausal women are the prevalent presentations [2,3]. In our study, 60% of the patients presented with abnormal uterine bleeding, which is in line with the findings from other studies [3,7]. Studies have found that many patients with lipoleiomyoma have metabolic derangements in the form of diabetes, hyperlipidemia, hypertension, and cholestasis [6]. A similar observation was reproduced in this study, as 55% of the patients had diabetes. Elevated serological markers have not been detected in isolated lipoleiomyoma, although there is documentation of elevated CA-125 in a patient with a giant lipoleiomyoma in the literature [2]. The common clinical diagnosis in symptomatic patients is leiomyoma. Occasionally, a giant subserosal lipoleiomyoma can be mistaken for an adnexal mass; ergo, lipoleiomyoma must be kept in the differential diagnosis when investigating a solid-cystic, presumed adnexal mass with an adipocytic component [1,9,10].

Radiology

Radiological investigations play a crucial role in preoperative diagnosis by elucidating the origin (uterine vs pelvic vs ovarian), nature (degenerative changes vs lipomatous neoplasm), and malignant potential. On ultrasound, they are seen as a hyperechoic, avascular mass with a hypoechoic rim delineating them from the adjacent myometrium. Vascularity has been documented in broad ligament fibroids [11]. Computed tomography imaging identifies this entity as a well-defined uterine mass with fat attenuation, but it falls short in delineating the origin, i.e., lipomatous tumors of the uterus from those of the ovary [3]. MRI, with its T1-weighted, T2-weighted, and fat-suppression sequences, is regarded as the most sensitive and specific modality. It can better help in confirming the origin, elucidating the adipocytic component, and excluding malignant features such as invasion, irregular margins, and necrosis. The presence of non-uniformity in the distribution of fat aligns more with lipomatous degeneration [2,8]. Lipoleiomyomas tend to occur as solitary lesions, with few documented cases of multiplicity [3]. All but one case in our study were solitary in occurrence. Many times, the lipomatous component is addressed only in postoperative histopathological reports, with a lack of radiological and gross insight. In our study, 38.8% of the cases were radiologically detected and 33% were identified grossly, exemplifying the above disadvantage. Macroscopically, they are well-circumscribed, yellowish tumors located intramurally in the posterior aspect of the uterine body [2]. This is in concurrence with this study as well, albeit two had their origin in the cervix. Microscopically, they are composed of smooth muscle fibers and fibrous tissue arranged in fascicles, intermixed with adipocytes. Occasional myxoid and hyaline degeneration can be seen. Epithelioid morphology, nuclear atypia, and the presence of lipoblasts warrant designation as atypical/bizarre lipoleiomyomas. Nuclear pleomorphism, increased mitoses, necrosis, or the presence of lipoblasts are absent in lipoleiomyoma. The presence of the aforementioned should alert to malignant transformation. By immunohistochemistry, the adipocytic component is positive for S100 and vimentin and weakly positive for smooth muscle actin (SMA), with a low Ki-67 labeling index.

Pathogenesis

The presence of adipocytes within a uterine myometrial neoplasm has been a baffling query, given the inherent absence of fatty tissue normally. This query paved the way for multiple potential theories of pathogenesis. It was initially thought to arise from embryonic rests in the uterus, serosal fat creeping into the myometrium during surgical procedures, or adipocytic degeneration of an existing leiomyoma [1]. The current operational pathogenesis is that these lesions arise from mesenchymal stem cells or progenitor cells that exist in the uterus [6,12]. This mesenchymal stem cell theory is supported by the positivity of CD34 in lesional spindle cells, which is a marker of uterine leiomyoma progenitor cells. The other proposed mechanism is metaplasia of spindle cells of the uterine myometrium or of the neoplastic spindle cells of leiomyoma [3]. A study conducted by Akbulut et al., using immunohistochemistry for Ki-67, ER, PR, and HMB-45, found these adipocytic cells to be ER- and PR-positive, with a higher Ki-67 labeling index. ER and PR positivity affirmed a female genital tract origin, and the higher Ki-67 labeling index aligned with a neoplastic process rather than a degenerative change [7].

Role of Estrogen

Estrogen and progesterone have been implicated in the proliferation of leiomyoma, whilst the contrary has been observed in lipoleiomyoma. Studies have found decreased expression of the ER by immunohistochemistry in the adipocytic component when compared to adjacent myometrium, attesting to an estrogen-deprived environment favoring the proliferation of this adipocytic element. This observation explains the proclivity for menopausal and perimenopausal women and their coexistence with metabolic abnormalities such as hyperlipidemia, hypothyroidism, diabetes, and hypertension, which are prevalent in this group of women with deranged estrogen metabolism [13]. Lipoleiomyomas have been observed to coexist with other gynecological pathologies such as adenomyosis, endometriosis, and endometrial hyperplasia, which is in line with our findings [2]. They have also been found incidentally while evaluating other gynecological malignancies arising from the endometrium, ovary, and cervix. The reverse was observed in one of our cases, wherein a hysterectomy done in view of a uterine mass, which turned out to be a lipoleiomyoma, resulted in the incidental discovery of cervical adenocarcinoma. Some of the explanations for these observations are as follows. Increased age allows for the accumulation of genetic mutations, resulting in neoplastic transformation, exemplifying the presence of malignancies in patients with lipoleiomyoma, which also tend to occur in the older age group. The metabolic risks in patients with lipoleiomyoma, in the form of excess local estrogen from conversion of androgen to estrogen through aromatization by the adipocytes of this neoplasm, combined with observed insulin resistance leading to increased IGF-1 signaling, create a pro-tumorigenic milieu for the development of malignancies [14,15]. However, there is no defined theory that implicates lipoleiomyoma as a risk factor for malignancy.

Treatment

Studies suggest expectant management for lipoleiomyoma, similar to leiomyoma, as they are benign and fertility is not a concern in the majority of patients. Surgeries such as hysterectomy or myomectomy, tumor embolization, radiofrequency ablation, and myolysis are considered based on tumor size, symptoms, surgical fitness of the patient, as well as availability of equipment [2,8]. Radiologically guided core needle biopsy in indeterminate cases helps avoid radical surgeries with significant postoperative morbidities. Hysteroscopy- or laparoscopically guided surgical resection also helps in reducing the above demerit. Hysteroscopy-guided procedures are useful in the resection of submucosal lesions, with the caveat of iatrogenic perforation. This is due to similarities between omental fat and the lesion of interest on scopic imaging. Few studies report malignant transformation and vascular metastasis, warranting close follow-up of these lesions [8]. It has been observed that the size of lipoleiomyoma increases at a rapid rate compared to other variants, given its estrogen independence, resembling a malignancy [16]. On follow-up, the rate of increase in size should be noted. One must also look out for tumor-to-tumor metastasis when they coexist with any other malignancies, as it has been documented in the literature [3].

Limitations

There are a few limitations to this study. It was a single-center study with a limited sample size. A multi-center study would have enabled a study of a larger, heterogeneous population, providing better insight into the precise nature of this entity. This study has found lipoleiomyoma to coexist with metabolic derangements such as diabetes mellitus and other gynecological pathologies, ranging from benign to malignant. It does not aim to give insight into pathogenesis or elucidate the pathophysiology behind the observed correlations. This study, being retrospective and descriptive in nature with limited statistical analysis, does not suffice to establish a causal relationship between the observed associations. Clinical follow-up of these cases may be assessed to design a more appropriate protocol of management.

## Conclusions

Given these associations, it is imperative to carefully evaluate patients clinically and pathologically for coexisting gynecological conditions and metabolic disorders when lipoleiomyoma is detected. Clinical vigilance is advised to avoid the delayed detection of potentially morbid and fatal diseases.
